# The managed clearing: An overlooked land-cover type in urbanizing regions?

**DOI:** 10.1371/journal.pone.0192822

**Published:** 2018-02-12

**Authors:** Kunwar K. Singh, Marguerite Madden, Josh Gray, Ross K. Meentemeyer

**Affiliations:** 1 Center for Geospatial Analytics, North Carolina State University, Raleigh, North Carolina, United States of America; 2 Department of Forestry & Environmental Resources, North Carolina State University, Raleigh, North Carolina, United States of America; 3 Center for Geospatial Research, Department of Geography, University of Georgia, Athens, Georgia, United States of America; University of Maryland at College Park, UNITED STATES

## Abstract

Urban ecosystem assessments increasingly rely on widely available map products, such as the U.S. Geological Service (USGS) National Land Cover Database (NLCD), and datasets that use generic classification schemes to detect and model large-scale impacts of land-cover change. However, utilizing existing map products or schemes without identifying relevant urban class types such as semi-natural, yet managed land areas that account for differences in ecological functions due to their pervious surfaces may severely constrain assessments. To address this gap, we introduce the managed clearings land-cover type–semi-natural, vegetated land surfaces with varying degrees of management practices–for urbanizing landscapes. We explore the extent to which managed clearings are common and spatially distributed in three rapidly urbanizing areas of the Charlanta megaregion, USA. We visually interpreted and mapped fine-scale land cover with special attention to managed clearings using 2012 U.S. Department of Agriculture (USDA) National Agriculture Imagery Program (NAIP) images within 150 randomly selected 1-km^2^ blocks in the cities of Atlanta, Charlotte, and Raleigh, and compared our maps with National Land Cover Database (NLCD) data. We estimated the abundance of managed clearings relative to other land use and land cover types, and the proportion of land-cover types in the NLCD that are similar to managed clearings. Our study reveals that managed clearings are the most common land cover type in these cities, covering 28% of the total sampled land area– 6.2% higher than the total area of impervious surfaces. Managed clearings, when combined with forest cover, constitutes 69% of pervious surfaces in the sampled region. We observed variability in area estimates of managed clearings between the NAIP-derived and NLCD data. This suggests using high-resolution remote sensing imagery (e.g., NAIP) instead of modifying NLCD data for improved representation of spatial heterogeneity and mapping of managed clearings in urbanizing landscapes. Our findings also demonstrate the need to more carefully consider managed clearings and their critical ecological functions in landscape- to regional-scale studies of urbanizing ecosystems.

## Introduction

Studies of human-modified ecosystems often rely on existing land use and land cover (LULC) products, such as the U.S. Geological Survey (USGS) National Land Cover Database (NLCD) [1, 2], or datasets that were produced using generic LULC classification schemes [3]. Utilizing existing products or schemes are useful for many science questions related to land change, but general-purpose LULC schemes may fail to adequately represent ecological functions that are important for particular studies, and/or may altogether omit important classes. For example, land uses such as road right-of-ways and utility lines that are pervious, yet regularly managed by mechanical clearing and herbicide applications, are often labeled as ‘developed’ in many classification schemes. The notion of developed implies impervious surface and the possibility of redevelopment, infill development, and future change; contrary to the characteristics of these semi-natural land-cover types and their contribution to ecosystem services. Therefore, LULC data without meaningful land cover types that differentiate ecological functions may imperil our efforts to achieve realism in urban ecosystems studies. Unfortunately, our present understanding of managed clearings is insufficient and represents an under-explored opportunity that could benefit realistic assessments of urbanizing ecosystems.

We define managed clearings as semi-natural vegetated land surfaces with varying degrees of management practices. Common examples of managed clearings are parks, golf course fairways and greens, recreational sports complexes, lawns, lands under electrical transmission lines, utility corridors, grassy and/or treed medians, and shoulders along roads, including water retention ponds. These, in aggregate, represent an important urban-rural component of interest due to their ability to alter ecosystem function (e.g., sequester different amounts of carbon, recharge groundwater, host different plant and animal species assemblages, and influence stormwater run-off), provide cultural value (e.g., recreational green spaces), and support human welfare (i.e., offer safe shoulders for highways or prevent interference with overhead electrical wires). The low possibility of being further developed into impervious uses sets managed clearings apart from other semi-natural land uses within the urban matrix. However, despite accounting for a substantial portion of urban landscapes and contributing considerably to ecosystem services [4], these areas are either not represented explicitly as an ecological functional class type in LULC classification schemes or they are counted among less specific land-cover classes that obscure their unique ecological function [5]. For example, both the NLCD classification scheme and the High Ecological Resolution Classification for Urban Landscapes and Environmental Systems (HERCULES) model assign urban land cover to various intensities of developed land (e.g., open space, low, medium and high intensity) or types (e.g., coarse and fine-textured vegetation), respectively [6, 7]. While a high proportion of sub classes in these land-cover types are pervious, they are labeled as developed, which implies the possibility of state change contrary to the characteristics of managed clearings [5]. Such land-cover types also affect area proportion in the study systems that might lead to erroneous outcomes. For example, Julian and Gardner (1) used a ‘> 50% threshold’ criterion to categorize watersheds into urban, agriculture, and forest types using NLCD data to analyze the effects of LULC on runoff patterns. Their findings suggests that urban watersheds were flashier and had less hydrologic memory compared to forest watersheds. However, the lack of specificity in the classification schema may lead to the same overall proportions of “developed space” that include different amount of impervious surface, which may have artificially increased the flashiness of storm water runoff in urban watersheds. Similarly, an inclusion of managed clearings at the county level may have provided an improved spatially explicit population projection model given that it represents low transition possibilities [8]. Any approach to LULC mapping that does not include managed clearings, therefore, may inadequately address urban ecosystem problems.

**Fig 1:**
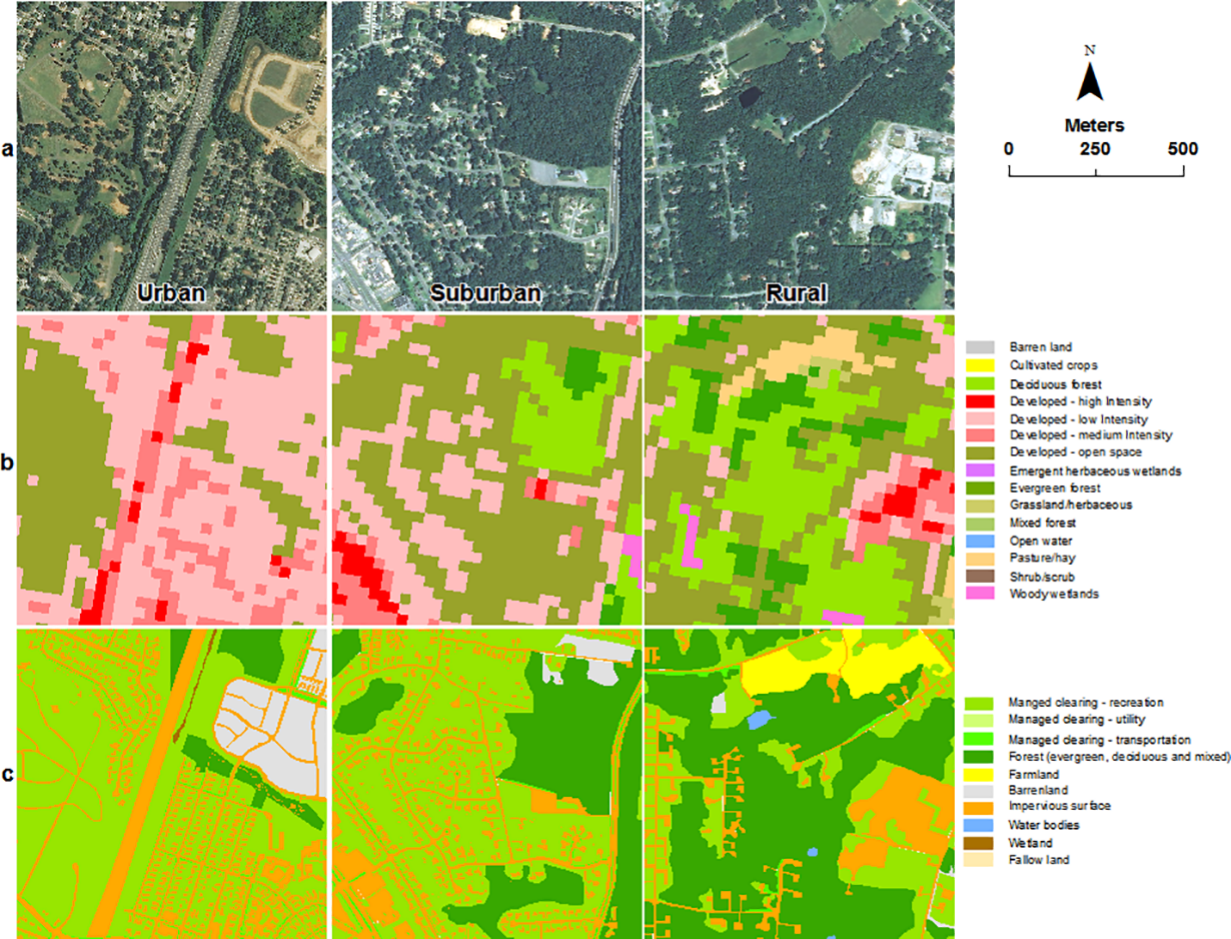
Managed clearings land-cover type. (a) The 2012 National Agriculture Imagery Program (NAIP) imagery for a detail view of land use and land cover across urban-rural gradients. (b) Land cover derived from the 2011 National Land Cover Database (NLCD) data. (c) NAIP-based hand-digitized land-cover data.

Urban open and green spaces are commonly used land cover type in urban ecosystem studies [9–12]. These land surfaces are undeveloped and accessible to the public [13], but they do not include all managed surfaces (e.g., lands under electrical transmission lines, utility corridors, grassy and/or treed medians, and shoulders along roads) and they rarely cross urban boundaries compared to managed clearings. When mapping urban landscapes, studies often overlook issues of what constitutes these land-cover types, their boundaries and spatial distribution, how to map these land-cover types, and type of management practices. Therefore, mapping managed clearings would be relatively harder due to its definition—any pervious and vegetated land surfaces with varying degrees of management practices—compared to any general-purpose LULC classification schemes. Besides, the spatial structure of managed clearings is comprised of varying sizes with irregular shapes, interspersed with natural and built-up surfaces at finer spatial scales (Fig 1). This makes high-resolution remote sensing data (i.e., between 1 and 5-m spatial resolution) an ideal choice for mapping managed clearings. However, issues such as the scale, cost, and spatial-temporal coverage leave us with fewer options for cost-effective, high-resolution data [14]. Examples include freely available U.S. Department of Agriculture (USDA) National Agriculture Imagery Program (NAIP) image data, DigitalGlobe imagery (i.e., IKONOS and QuickBird), and European Space Agency (ESA) Sentinel images. Characteristics such as the 1-meter spatial resolution and the availability of a near-infrared band make NAIP data a good choice for mapping urbanizing landscapes among the high-resolution remote sensing data, in particularly, using object-based image analysis (OBIA) and visual interpretation [15]. OBIA is a process of partitioning remote sensing imagery into meaningful image-objects by incorporating spectral, spatial and expert knowledge. This method is a popular choice for mapping urban landscapes [16–18]. However, success of OBIA often relies on the availability of specialized software, high-resolution imagery of consistent illumination, and the existence of expert knowledge and familiarity with targeted landscapes [19].

Here, we introduce the concept of managed clearings through a case study focused on the Atlanta, GA, Charlotte, NC, and Raleigh, NC area (known collectively as the “Charlanta” megaregion). We addressed the following research questions: How abundant are managed clearings relative to other land-cover types, and how does the area of managed clearings differ between metropolitan areas? How do recreational, transportation, and public utility services in managed clearings differ in areal proportion? Finally, we compared our managed clearing mapping outcomes with NLCD data for addressing the following questions: Can we modify the NLCD data to accurately represent the amount of managed clearings in urbanizing landscapes, and if yes, how does the proportion of manually interpreted NAIP-derived managed clearings compare to the NLCD’s developed land-cover types?

## Materials and methods

### Study area and data sources

**Fig 2:**
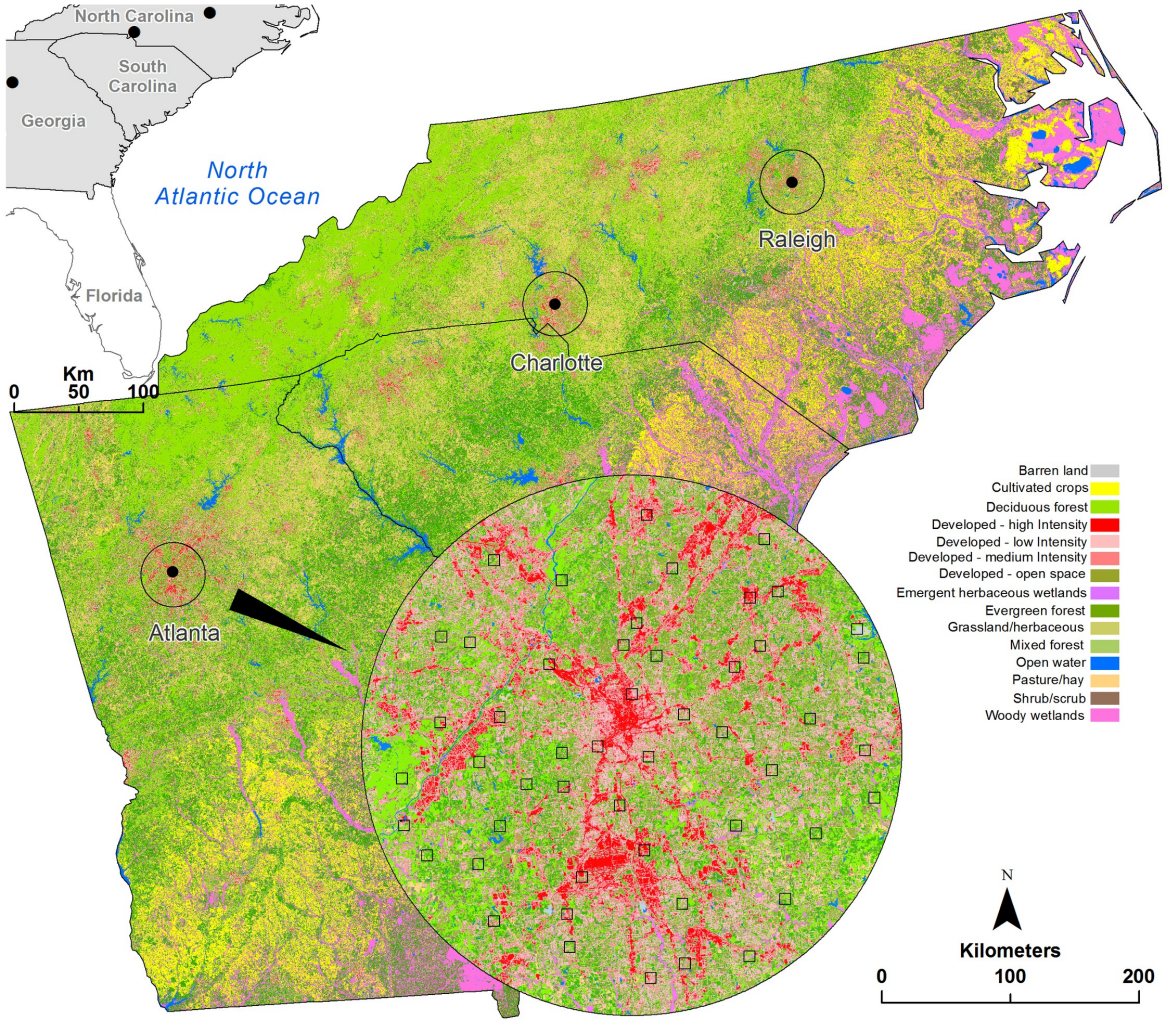
Study area. 2011 National Land Cover Database with an overlay of a 25 km buffers around the center of Atlanta (Georgia), Charlotte and Raleigh (North Carolina) in the Charlanta megaregion in the southeastern United States.

Charlanta is the third largest megaregion in population and economy in the United States. It is located within the Piedmont physiographic province between the Appalachian Mountains and the Atlantic coastal plain (Fig 2). The region’s rolling landscape has some of the highest densities of secondary and tertiary road networks in the United States and has few environmental obstacles for automobile dependent growth [20]. The megaregion has over 6% of the total U.S. population, and it is rapidly growing with a 24% increase expected between 2010 and 2025. Development in Charlanta is characterized by sprawling urbanization with low- to medium-density housing that has led to the conversion of forest and farmland dominated landscapes to other land uses and highly fragmented urban forests [20].

We used two data sources: high-resolution NAIP 2012 aerial imagery, and NLCD 2011 data, acquired from the United States Department of Agriculture and USGS, respectively. The 1-meter spatial resolution of NAIP imagery corresponds to the spatial heterogeneity of typical suburban development and reflects fine-scale impacts on ecological phenomena. We selected 2012 NAIP imagery over three-band 2011 NAIP due to the presence of a near-infrared band (i.e., blue, green, red, and near infrared band). The four-band NAIP images were acquired in the Universal Transverse Mercator coordinate system (NAD 83, meters). We reprojected the 2011 NLCD data to the same coordinate system. The NLCD data uses a 16-class LULC classification scheme [21] and is primarily based on a decision-tree classification of Landsat data with a spatial resolution of 30 meters [7].

### Classification schemes for mapping managed clearings in urbanizing landscapes

We used the Anderson classification scheme level I [22] and divided the ‘urban or built-up’ class into impervious surfaces and managed clearings categories (hereafter referred to as “modified classification scheme”). The Anderson scheme is a flexible hierarchical classification system suitable to capture the level of detail and scale required by a mapping application [23]. It is also the base classification system for NLCD data. We selected the level I scheme to modify the urban or built-up class for (1) mapping urban-rural gradient based on ecological functions rather land-cover intensity and uses, and (2) comparing NAIP-derived LULC to NLCD data. We further subdivided the managed clearings class into three categories of recreation, transportation, and utility types to account for varying management practices and their areal proportion. Recreational managed clearings are highly managed may covered with either grass, tree or both such as parks, lawns, recreational sports complexes, and golf courses. ‘Transportation’ included grassy and/or treed medians, pervious sidewalks, and shoulders along roads whereas the ‘utility’ type included lands under electrical transmission lines. These managed clearings are subject to minimal to high management practices and have a low chance of changing in the future (Table 1).

**Table 1:** Anderson classification scheme and the description for the modified classification scheme, including sub classes of managed clearings.

Anderson classification scheme level 1	Modified classification scheme	Description
Urban or built-up land	Impervious surfaces	Intensive use areas characterized by impervious structures
	Managed clearings—recreation	Highly managed with low to moderate possibility for change such as parks, lawns, recreational complexes, golf course, and greens.
	Managed clearings—transportation	Moderately managed with low to no chance for change such as grassy and treed medians and shoulders along roads, etc.
	Managed clearings—utility	Almost no management practices and with little chance for change, such as lands under electrical transmission lines, flood plains, etc.
Agricultural land	Farmland	Croplands and pastures
Rangeland ^†^	Fallow land	
Forest land	Forest	Deciduous, evergreen, mixed, and managed forests
Water	Water	Streams, canals, lakes and reservoirs
Wetland	Wetland	
Barren land	Barren land	Areas of exposed soil, sand or rock
Tundra ^†^		
Perennial Snow or Ice^†^		

†Not present in study system.

### Mapping managed clearings land cover

We used visual image interpretation to manually digitize LULC features including managed clearings from the NAIP imagery due to its better performance with higher accuracy estimates compared to other classification methods, including OBIA [24]. Although manual interpretation of LULC from aerial imagery has been performed for over 75 years and is known to result in datasets with higher thematic accuracy, it requires substantial levels of effort and time to complete the mapping. To overcome this challenge, 50 randomly distributed 1-km^2^ blocks were established within the 25-km radius around each city center to ensure adequate representation of urban, suburban and rural landscapes. We manually interpreted and digitized LULC for the three cities for each sample block. To map and differentiate forest from managed clearings that contained trees, we used a half-acre (~2000 m^2^) minimum mapping unit (MMU) and landscape context. We tallied the total area of each land-cover type for each 1-km^2^ block, and then tabulated areas for all 50 blocks.

### Modification of NLCD data

We repeated the same method for the NLCD data by estimating the total area of each land cover type for each 1-km^2^ block, and then tabulated area for all 50 blocks of Atlanta, Charlotte, and Raleigh (Table 2). Further, LULC estimates were modified for each block of the study area into categories suitable for comparison with the manually interpreted NAIP data and to explore the possibility of modifying NLCD data for representing managed clearings. We combined similar LULC types into broader classes, such as the forest types (e.g., deciduous, evergreen, and mixed) into one forest category. We also combined subcategories of the developed types in two ways to represent managed clearings using (1) the similarity between subcategories, and (2) the percent imperviousness in each subcategory. For the first aggregation, we combined low-, medium-, and high-intensity into the impervious class and renamed developed open-space to managed clearings. For the second aggregation, we combined the percent impervious proportion of the developed (e.g., open, low, medium and high intensity) types into the impervious class and merged the remaining proportion with herbaceous land cover to create managed clearings. The NLCD classification states that the developed low-intensity is comprised of up to 75% open, green space, and developed medium-intensity represents as much as 50% green space. Therefore, aggregating these two classes with the developed high-intensity into a redefined impervious land cover may not reflect the true nature of the NLCD classification system. To address this, we also compared NAIP-derived managed clearings to the NLCD classification without any changes. This resulted into three comparisons that explore the possibilities for modifying NLCD data to represent managed clearings.

**Table 2:** Land use and land cover estimates based on 50 randomly selected 1-km^2^ segments across the urban-rural gradients within the 25-km radius around each city center of Atlanta, Charlotte, and Raleigh using the National Land Cover Database.

Land cover	Code and description	Area (km^2^)		
		Atlanta	Charlotte	Raleigh
Water	11—Open water	0.22	1.69	0.65
Developed	21—Developed, open space^†^	12.03	12.14	13.03
	22—Developed, low intensity^†^	9.73	8.59	6.19
	23—Developed, medium intensity^†^	5.75	3.76	2.92
	24—Developed, high intensity^†^	4.28	1.53	0.76
Barren	31—Barren land (rock/sand/clay)	0.58	0.30	0.19
Forest	41—Deciduous forest	8.18	11.83	8.18
	42—Evergreen forest	6.30	3.36	5.61
	43—Mixed forest	0.50	0.19	2.18
Shrubland	52—Shrub/Scrub	0.49	0.62	0.58
Herbaceous	71—Grassland/herbaceous	0.70	1.59	2.29
Planted	81—Pasture/hay	0.58	3.74	4.67
	82—Cultivated crops	0.00	0.02	1.36
Wetlands	90—Woody wetlands	0.59	0.59	1.37
	95—Emergent herbaceous wetlands	0.05	0.06	0.02
Total		50.00	50.00	50.00

^†^ Distribution of percentage imperviousness among developed land-cover types (Open space—<20%; low intensity– 20–49%; medium density– 50–79%; and high-density– 80–100%) in NLCD data.

## Results

### Distribution of managed clearings in NAIP-derived LULC data

Assessment of manually digitized NAIP imagery revealed managed clearings as the second most dominant cover type after forest at both city and regional extents (Table 3). Managed clearings covered 28.5% of the total sampled areas of Charlanta megaregion, 6.3% higher than the total impervious surface area and 12% lower than the forest cover. In terms of cumulative pervious surface, managed clearings when combined with forest cover constitutes 69% of pervious area in the region with varying degrees of vegetation. Total area of managed clearing varied slightly across the three cities from the minimum 24% in Raleigh, NC, to a maximum of 31.5% in Atlanta, GA. The managed clearings—recreation type comprised up to 90% of the sampled area in each city followed by transportation and utility subtypes at 2% and 1% of the total mapped LULC of Charlanta (Table 3).

**Table 3:** Land use and land cover estimates across the urban-rural gradients of Atlanta, Charlotte, and Raleigh based on visual interpretation of 2012 National Agriculture Imagery Program imagery.

Modified classification scheme	Area (km^2^)			
	Atlanta	Charlotte	Raleigh	Total
Impervious surfaces	14.05	9.52	9.76	33.33
Managed clearings—recreation	14.24	12.89	10.75	37.88
Managed clearings—transportation	0.84	1.20	1.04	3.08
Managed clearings—utility	0.66	0.71	0.31	1.68
Farmland	0.08	2.49	3.58	6.15
Fallow land	0.57	0.51	0.65	1.73
Forest	17.86	20.38	22.47	60.71
Water	0.42	1.57	0.82	2.81
Wetland	0.17	0.23	0.34	0.74
Barren Land	1.08	0.50	0.29	1.87
Total	50.00	50.00	50.00	150.00

### Land use/land cover disparity between classification schemes

**Fig 3:**
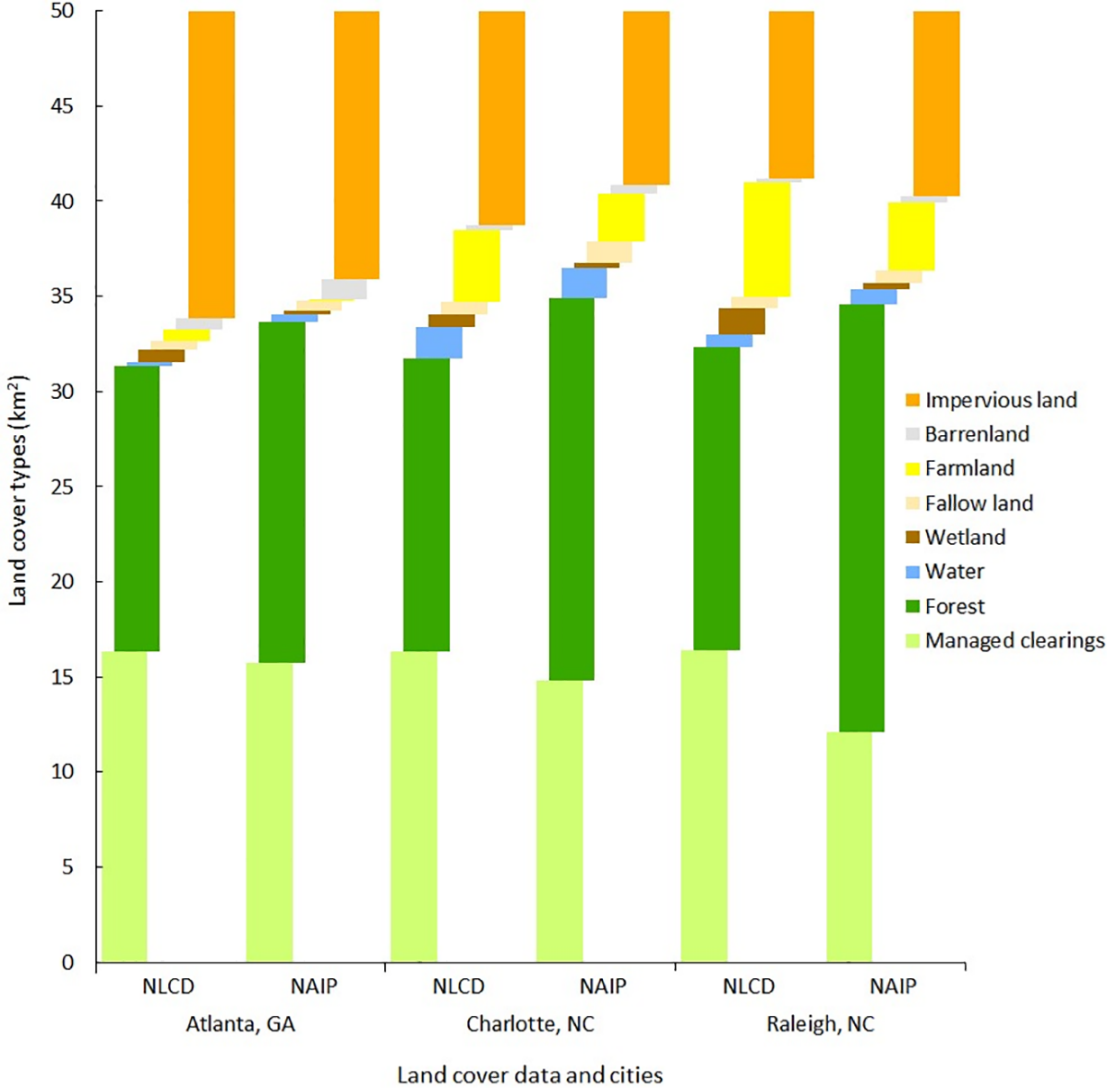
Proportions of mapped land use and land cover types derived from the National Land Cover Database (NLCD) and the visual interpretation of National Agriculture Imagery Program (NAIP) imagery.

We observed a substantial variation in the total area of managed clearings in three comparisons of manually digitized 1-m NAIP images and 30-m NLCD. The total area of managed clearings from NAIP imagery was higher than the combined subcategories (i.e., LULC similarity) of the NLCD developed type by 5.5 km^2^ while area of impervious surfaces was lower by 10 km^2^ (Fig 3). Forest was the most dominant land cover in both data sets (40.5% and 30.9%, respectively) followed by managed clearings in NAIP-derived data (28.5%) and impervious surfaces in NLCD data (29%) (Table 4). NAIP-derived managed clearings and impervious surfaces were 6.5 km^2^ and 2.9 km^2^ lower, respectively, compared to NLCD data created using percentage impervious (Fig 3). We observed a higher total managed clearing area at both city and regional extents (5.5 km^2^) using NAIP imagery compared to the original NLCD developed land-cover types. The total area of impervious land cover was also higher. Apart from these variations, the total areas of forest, open water, and barren land from the NAIP-derived data were higher than the NLCD data with net gains of 14.5 km^2^, 0.3 km^2^, and 0.8 km^2^, respectively. Table 4 illustrates the net change (gains and losses) in mapped areas from manually digitized NAIP imagery and NLCD data. Overall, none of these comparisons showed areal proportions equal to visually interpreted LULC data so, adapting NLCD data would not work to represent managed clearings in urban landscapes.

**Table 4:** Comparison of total land use and land cover estimates and net change (gain or loss) between the NAIP-derived data and an eight-class NLCD scheme for Atlanta, Charlotte, and Raleigh. We merged developed low, medium, and high intensity into the impervious class and changed developed open-space to managed clearing, and combined the percent impervious proportion of the developed types into the impervious class and merged the remaining proportion with herbaceous land cover to create managed clearings. Missing values indicate no comparison.

Data aggregation method	NLCD classification scheme	Modified classification scheme	Atlanta (km^2^)			Charlotte (km^2^)			Raleigh (km^2^)		
			NLCD	NAIP	Net	NLCD	NAIP	Net	NLCD	NAIP	Net
Land use and land cover similarity	Developed†	Impervious land	19.76	14.05	5.71	13.87	9.52	4.35	9.87	9.76	0.11
	Developed, open space	Managed clearings	12.03	15.76	-3.73	12.14	14.79	-2.66	13.03	12.10	0.94
Percent impervious	Impervious land*	Impervious land	16.15	14.05	2.10	11.26	9.52	1.74	8.80	9.76	-0.96
	Managed clearings**	Managed clearings	16.34	15.76	0.58	16.35	14.79	1.56	16.39	12.10	4.29
Original developed land-cover types	Developed, open space	Managed clearings	12.03	15.76	-3.73	12.14	14.79	-2.65	13.03	12.10	0.93
	Developed, low intensity	-	9.73	-	-	8.59	-	-	6.19	-	-
	Developed, medium intensity	-	5.75	-	-	3.76	-	-	2.92	-	-
	Developed, high intensity	Impervious land	4.28	14.05	-9.77	1.53	9.52	-7.99	0.76	9.76	-9.00
Forest		Forest	14.98	17.86	-2.84	15.38	20.38	-5.00	15.97	22.47	-6.50
Open water		Water	0.22	0.42	-0.20	1.69	1.57	0.11	0.65	0.82	-0.17
Wetlands		Wetland	0.64	0.17	0.47	0.65	0.23	0.42	1.39	0.34	1.05
Shrubland		Fallow land	0.49	0.57	-0.08	0.62	0.51	0.11	0.58	0.65	0.07
Farmland		Farmland	0.59	0.08	0.51	3.76	2.49	1.27	6.03	3.58	2.45
Barren land (rock/sand/clay)		Barren land	0.58	1.08	-0.50	0.30	0.50	-0.20	0.19	0.29	-0.10

*A combination of percentage impervious surfaces of NLCD developed land-cover types

**A combination of percentage pervious surfaces of NLCD developed and herbaceous land-cover types

†A combination of low, medium and high development intensity land-cover types

NLCD—national land cover database, and NAIP—national agriculture imagery program

### Developed versus managed clearings land-cover types

The cumulative area of the four sub-categories of NLCD’s developed land-cover type constituted the most dominant land-cover type and covered over 50% of the area in each city and regionally (Tables 2 and 4). When we excluded the developed open-space type, the area proportions dropped to 40%, 28% and 20% (Atlanta, Charlotte and Raleigh, respectively), which is still approximately 28%, 9% and 1% higher than impervious surfaces mapped using NAIP imagery with the modified classification scheme (Table 4).

## Discussion

Managed clearings, such as lawns, public parks, and highway medians, are an integral part of urban and suburban landscapes. As the name implies, these areas require management in the form of mowing, tree/shrub removal, and/or application of herbicides and other maintenance in order to remain in a relatively cleared state. LULC data for urban landscapes that does not account for managed clearings may limit our ability to adequately model and address human-modified ecosystem problems, in turn leading to erroneous or misguided policies and management practices. In this study, we investigated the abundance of managed clearings relative to other land-cover types using NAIP imagery, and we compared this to the proportion of land-cover types in NLCD data. Managed clearings in NAIP-derived data is the second most dominant land-cover type after forest and comprises more than one-fourth of the total mapped area in the Charlanta megaregion. We observed a large variation in area estimates between NAIP-derived LULC data and NLCD data suggesting the use of high-resolution remote sensing data for mapping managed clearings versus modifying NLCD data. These findings validate our assumption that a higher proportion of maintained, ecologically functional, pervious managed clearings exist in urbanizing landscapes with inadequate representation in various standard data products and traditional classification schemes. Substantial differences in total land-cover areas between digitized 1-m NAIP images and 30-m NLCD data, while not surprising [25, 26], do suggest including managed clearings in urban landscape mapping to better capture ecologically functional land-cover types.

Area estimates derived from NAIP data revealed managed clearings to be the second most dominant land-cover type after forest within the Charlanta megaregion and in each of the three mapped cities. This essentially rules out a coincidental pattern of dominance among other land-cover types in urbanizing landscapes. In a similar study, Singh *et al* [27] observed managed clearings as the most dominant cover type in the urban and suburban areas of Charlotte, covering more than 38% of the total area. In the Singh *et al* [27] study, the dominance of managed clearings was perhaps due to mapping the entire Mecklenburg County compared to limiting the area of this study within a 25-km radius buffer from the city center using 50 randomly selected 1-km^2^ blocks. Typically, areas away from urban centers contain a much higher quantity of managed clearings. For example, land-cover types such as lawns, grassy and treed medians, and shoulders along roads are more dominant in suburban and exurban areas adjoining the urban centers. Nonetheless, whether the mapping of urban landscapes is wall-to-wall or within a set of randomly distributed sample areas, the prevalence of managed clearings in urbanizing landscapes is substantial. The area of managed clearings in the Charlanta megaregion was 4.3% higher (~ 6.5 km^2^) than the developed open-space category in NLCD data, which, as per the NLCD classification scheme definition, may contain up to 20% impervious surface of total land cover [21]. This difference is critical since managed clearings are completely pervious and maintained, and mapped area estimates are based on sampled plots.

We expected the proportion of managed clearings-transportation to be similar to the managed-clearings-recreational for two reasons: First, we regularly visualize grassy and/or treed medians and shoulders along roadways during our daily commutes, which gives us a greater sense of prominence of these managed clearings in urban landscapes. Second, managed clearings-transportation are easily distinguishable from other land-cover types due to their pattern, shape and juxtaposition with other land-cover types. However, our findings reveal the transportation sub-category as the second most dominant with far less in area proportion than the managed clearing- recreational type. This finding could change with wall-to-wall mapping and may vary regionally as management practices differ for each urban region. We also observed difficulties in identifying and mapping utility lands, with the exception of lands under electrical transmission lines. Therefore, the area proportion of managed clearings-utility will likely also change if we use *a priori* knowledge of their existence in urban regions. Our proposed subcategories of managed clearings can help assess the impacts of management practices, such as mowing and herbicide use, as well as storm water infiltration, on urban ecosystem services, thus we require establishment of guidelines for accurate mapping and differentiation of these managed clearing types.

The 1-m spatial resolution of NAIP imagery allows us to differentiate fine-scale variations among managed clearings, tree canopy, and impervious surfaces of urban landscapes compared to medium-resolution remote sensing data (e.g., Landsat) and products (e.g., NLCD data). We used a half-acre (~2000 m^2^) MMU and landscape context to map and differentiate among land-cover types, in particular, forest and managed clearings cover types. For example, we mapped a linear tree structure as managed clearings-transportation if it was located within medians along roads. Since we visually interpreted and digitized these land-cover types, we expected area estimates to be accurate and true representations of urbanizing landscapes. Regarding the variability in area estimates between NAIP and NLCD data, the MMU may have played a role in reducing area estimates of forest and managed clearings in the NLCD data. Nowak *et al* [25] observed lower canopy (small patches of trees) and impervious cover estimates from 2001 NLCD data compared to high-resolution photo-interpreted estimates of Google Earth imagery and attributed this difference to fine-scale variations in canopy that were not detected by the NLCD method. Wickham *et al* [28] and Maxwell *et al* [29] observed a similar pattern in their studies using NLCD data and attributed this to smoothing of the fine-scale variation within coarser resolution datasets. Nowak *et al* [25] suggested urban development would lead to increased impervious cover in future image dates and thus underestimation by NLCD data. These issues are much more prominent in mapping land cover in urban landscapes. Since we manually interpreted and digitized land-cover types from the NAIP imagery, we thus avoided issues Nowak et al [25] mentioned in their study such as high temporal variation (from the early to late 2000s) in photo-interpreted imagery. Therefore, it is worth noting that the NLCD data are suitable for regional scale studies, but could lead to unrealistic simulation and modeling of environmental issues [25] in urban areas due to inconsistent estimates of urban land-cover types. Overall, our comparison of land cover derived from NAIP imagery and NLCD data suggests that NLCD users cannot modify NLCD data to represent managed clearings. Furthermore, the modification of NLCD data beyond its stated classification description might be impractical.

Area estimates of managed clearings using NAIP imagery are substantially high (more than 25% area of all LULC types) and are thus considered to be important in LULC classification schemes for characterizing urban landscapes. Managed clearings are semi-natural land surfaces with varying degrees of management, and they are often spectrally similar to other vegetated (e.g., fallow and pasture land) and non-vegetated (e.g., exposed soil) surfaces. A critical question is how to classify NAIP or any other high-resolution remote sensing imagery for wall-to-wall mapping of managed clearings apart from using the visual interpretation? Due to spectral similarity among the managed clearings types, this would very difficult using automated digital classification methods. The OBIA classification may offer a promising solution to this challenge [30]. OBIA has increasingly been applied with high-resolution remote sensing data to produce wall-to-wall LULC maps of urbanizing landscapes [17]. However, two factors limit the use of OBIA in mapping managed clearings. First, the spectral signatures of the three managed clearings cover types are fundamentally similar due to the presence of mixed vegetation. Second, additional information such as the spatial shape, size, and pattern, are required for landscape context to identify and delineate managed clearings. In addition, OBIA works better with imagery from satellite platforms vs. airborne due to different illumination with different flight pattern over the study area. Based on this study, further exploration of the advantages and challenges of OBIA including managed clearings and subcategories in urban mapping is warranted, as well as a more careful consideration of how managed clearings function differently than other urban land covers is needed.

## Conclusions

Managed clearings are a semi-natural, vegetated and ecologically important land cover with varying degrees of management practices used to maintain their cleared and open status in urbanizing landscapes. Our results highlight managed clearings as a dominant land-cover type comprising more than one-fourth of the urban composition in our sample area. Our study also demonstrates the need to derive managed clearings using high-resolution remote sensing data for obtaining true representations of urbanizing landscapes. Because managed clearings have important functional differences (vegetated, pervious, managed) compared to the developed impervious surface urban categories in which they are usually aggregated, the addition of managed clearings to classification schemes is expected to improve urban growth predictions, ecological simulations, and modeling of urban ecosystems.

## Supporting information

S1 Dataset This zip folder contains the manually digitized National Agricultural Imagery Program (NAIP) imagery mapped land-cover types (shapefiles) and National Land Cover Database (NLCD) data (tiff) based on 50 randomly selected 1 km^2^ blocks across the urban-rural gradients of Atlanta, Charlotte, and Raleigh. Private Figshare link: https://figshare.com/s/b7ba65734bfa1ec8adbb
DOI: 10.6084/m9.figshare.5594629.
(ZIP)
